# Vietnamese people’s behavior and future intention to purchase medicines and functional foods on the internet: a cross-sectional study

**DOI:** 10.1038/s41598-024-75029-5

**Published:** 2024-10-16

**Authors:** Dung Anh Doan, Nhung Hong Vu, Phuong Lan Nguyen, Dai Xuan Dinh

**Affiliations:** 1 Faculty of Pharmacy, Phenikaa University, Hanoi, Vietnam; 2 Faculty of Pharmaceutical Management and Economics, Hanoi University of Pharmacy, 13-15 Le Thanh Tong, Hoan Kiem District, Hanoi City, Vietnam; 3 Drug Administration of Vietnam, Ministry of Health, Hanoi, Vietnam

**Keywords:** Online shopping, Online purchasing, Online buying, Behavior, Intention, Medicine, Dietary supplement, Functional food, Medical product, Internet, Public health, Medical research

## Abstract

This study investigated Vietnamese people’s behavior and future intention to purchase medicines and functional foods online and their associated factors. From March to May 2023, a Google Form questionnaire was distributed via social networks and online platforms. Convenience and snowballing sampling methods were employed to recruit 1,305 Vietnamese people. In the past year, 50.3% of participants purchased at least one kind of medicine and/or functional foods online (medicines: 27.6%, functional foods: 45.1%). Among 656 buyers, nearly a third bought these products more than three times and only 5.9% felt dissatisfied with their previous experiences. This purchasing behavior was more prevalent among females, those married, having higher education levels, usually shopping online, having longer time of Internet use per day, and seeking health information on the Internet (*p* < 0.001). In addition, 77.5% of participants intended to purchase these products online in the future and 61.2% would introduce this kind of online purchase to other people. The purchase intention was significantly associated with the participants’ previous experiences, area, contracting chronic diseases, and using the Internet for self-medication, while factors associated with the introducing intention included their education level, occupation, and previous experiences in online purchases (*p* < 0.001).

**Supplementary Information:**

The online version contains supplementary material available at 10.1038/s41598-024-75029-5.

## Introduction

In recent years, the rapid development of digital technology and the global Internet boom have given rise to a plethora of advantages for humanity. In the medical field, the roles of the Internet and social media are undisputable, especially in the context of the outbreak of pandemics and epidemics. The Internet has become a popular source for everyone to seek health information^1,2^. In the pharmaceutical field, the development of the Internet has contributed to providing pharmaceutical services to underserved areas, improving access to medicines, eliminating barriers for people with disabilities or senior citizens, and supporting people during lockdowns because of plagues^3^. However, the growth of the Internet and social networks also leads to many drawbacks. The ubiquity of electronic gadgets and easy access to the Internet facilitate inappropriate behaviors such as self-diagnosis and self-medication^4^. People (including children and adolescents) can easily buy medications online. More importantly, people can purchase counterfeit or substandard medicines that can have detrimental effects on their health^5^. Prescription-only medicines, such as antibiotics, can be bought without prescriptions^6,7^. Rogue or unapproved online pharmacies were also an intractable problem reported in many previous studies^8–10^. These issues contributed to the complexity of the online market.

Despite the potential risks mentioned above, many people still buy medical products online. Recently, the percentage of people purchasing these products has increased, especially during the COVID-19 pandemic^11–15^. In many countries, regulatory frameworks specific to online pharmacies and trading medicines on the Internet have yet to be enacted^7^. No legal documents have been promulgated in Vietnam to guide and manage these activities. In Vietnam, medicines and functional foods are two common types of products sold in pharmacies. As per the Law on Pharmacy, medicines are “preparations that contain active ingredients or herbal ingredients used for the prevention, diagnosis, treatment, alleviation of diseases in humans, and regulation of human physiological functions, including modern drugs, herbal drugs, traditional drugs, vaccines, and biologicals”^16^. Functional foods are foods used to support the function of parts of the human body, have nutritional effects, make the body comfortable, increase immunization, and reduce the risk of contracting diseases, including supplemented foods, health supplements/dietary supplements/food supplements, food for special medical purposes/medical food, and food for special dietary uses^17,18^. To distinguish from medicines, the packaging of functional foods must include the words “Functional food” and “This product is not medicine and cannot be replaced with medicine”^19^.

In the past, Vietnamese people had to go to pharmacies to purchase medicines and functional foods. The Internet and social networks have paved the way for trading these products online in recent years. People could buy medicines and functional foods over the Internet via websites, social networks, and mobile applications. The Ministry of Health and relevant organizations have endeavored to curb illegal activities involving the online trade of medicines^20^. However, this type of trade still blatantly happens. Many sellers have taken advantage of customers’ lack of knowledge, loopholes in laws, and the uncontrollability of the online market to trade these products on the Internet. Approximately 79.1% of Vietnamese people (78.44 million citizens) use the Internet^21^. This vast number of Internet users can become potential online buyers. However, no studies have been conducted to assess Vietnamese people’s online purchase of medicines and functional foods. This study was carried out to investigate Vietnamese people’s behavior and future intention to purchase medicines and functional foods online and their associated factors.

## Methods

### Study setting, sampling method, and data collection

The following formula was employed to estimate the minimum sample size for a proportion: n = Z_α/2_^2^.p.(1-p)/d^2^. With α = 0.01, Z_α/2_=2.575, *p* = 0.5 (to attain the maximum of p.(1-p)^22^), d = 0.05, the minimum sample size was 663 people. The research team endeavored to approach as many Vietnamese people as possible to increase the reliability, generalization, and extrapolation of results. The questionnaire was designed as a Google Form link and distributed to participants via social networks and online platforms (including Facebook, Messenger, and Zalo, a chatting app popularly used by Vietnamese people). The research team used convenience and snowballing sampling methods to recruit participants from March to May 2023. Eligible participants must be Vietnamese people, be able to understand Vietnamese, be at least 18 years old, and use the Internet.

### The questionnaire and variables

The research team developed a questionnaire after reviewing documents and referencing previous scientific articles^12–20,23–31^. Regarding face validity, a government official from the Vietnam Ministry of Health and two lecturers/researchers from the Phenikaa University and Hanoi University of Pharmacy reviewed the initial questionnaire. After that, a pilot study with the participation of 30 Vietnamese people was conducted to assess the clarity and intelligibility of each question and compute the questionnaire’s internal consistency. Two weeks later, these 30 participants answered this questionnaire one more time. Their responses between the two measurements were compared to evaluate the questionnaire’s test-retest reliability. The data of these people were not used in the final analysis. The final questionnaire (Vietnamese language) embraced four main parts: (1) participants’ characteristics, (2) Internet use, (3) knowledge/attitudes towards purchasing medicines/functional foods online, and (4) their purchase behavior and future intention. The definitions of medicines and functional foods (in the Introduction above) were also mentioned in the questionnaire to guarantee that participants could comprehend all questions.

### Primary outcomes/dependent variables

Regarding purchase behavior (eleven questions in total), three main questions included: (1) “*Have you ever purchased medicines online in the past year?*”, (2) “*Have you ever purchased functional foods online in the past year?*”, and (3) “*Have you ever bought medicines and/or functional foods online from foreign sources (such as international websites)?*”. A person was classified as “purchased medicines and/or functional foods” if this person answered “Yes” to either of the first two questions. Online buyers were also asked about (4) their level of satisfaction, (5) reasons for their satisfied or dissatisfied experience, (6) names of products purchased online, (7) the number of times they purchased medicines/functional foods online in the past year, (8) several activities when they purchased products online, (9) points of purchase, (10) how they knew the online purchase of medicines/functional foods, and (11) their criteria to choose the points of online purchase.

About future intention, three yes/no questions included: (1) “*In the future*,* will you purchase medicines on the Internet?*”, (2) “*In the future*,* will you purchase functional foods on the Internet?*”, and (3) “*Do you intend to introduce the online purchase of these products to other people (such as friends and family members)?*”. A person was classified as “having the intention to purchase medicines and/or functional foods in the future” if this person answered “Yes” to either of the first two questions.

### Independent variables

Participants’ knowledge and attitudes towards purchasing medicines and functional foods online were measured via 31 items (knowledge: 8 items and attitudes: 23 items). The items and scoring can be seen in Table S1. Participants’ scores could range from 0 to 16 (knowledge) and 23 to 115 (attitudes). The good reliability of this questionnaire was demonstrated via Cronbach’s alpha (knowledge: 0.85, attitudes: 0.95), Omega Total (0.96), and Spearman-Brown split-half reliability (knowledge: 0.82, attitudes: 0.94). In addition, its good test-retest reliability between two different times of measurements was displayed with intraclass correlation coefficients (ICCs) higher than 0.75 (knowledge: 0.80 (95% confidence interval (95%CI): 0.67–0.89), attitudes: 0.93 (95%CI: 0.90–0.96)).

Questions regarding participants’ Internet usage included (1) their frequency of Internet use (rarely, sometimes, usually), (2) average time of Internet use per day (hours), (3) frequency of online shopping for any products or services (never, rarely, sometimes, usually), (4) using the Internet to seek health information (yes/no), (5) self-diagnosis without visiting a doctor based on health information from the Internet (yes/no), and (6) self-medication without consulting a pharmacist or doctor based on health information from the Internet (yes/no). In addition, ten questions about participants’ general characteristics encompassed (1) year of birth, (2) sex, (3) residence/region (north, central, south), (4) area (urban, rural), (5) highest level of education, (6) marital status, (7) working status/occupation, (8) average monthly income, allowance, or retirement pension, (9) having a health insurance card, and (10) contracting chronic diseases.

### Data analysis

R software version 4.3.3 was used to analyze the data using the following packages: *psych*,* irr*,* ggplot2*,* gridExtra*,* BAS*,* ResourceSelection*,* Epi*, and *pROC*. Numeric variables (such as age) were described via mean and standard deviations (SD), while exact numbers and percentages were used to report categorical variables (such as sex). Factors associated with Vietnamese people’s behavior and intention to purchase medicines and functional foods online were identified via univariate and multivariate logistic regression models. To minimize the models’ complexity and avoid multicollinearity/overfitting, the Bayesian Model Averaging method was employed to select independent variables in multivariate models. Furthermore, Hosmer–Lemeshow tests and the area under the curve (AUC) were computed to assess the goodness of fit of these models. A p-value < 0.001 was considered statistical significance.

### Ethics approval and consent to participate

This study was approved by the Institutional Review Board of the Hanoi University of Pharmacy (No: 193/QĐ-DHN) and the ethics committee of the Nam Dinh University of Nursing (No: 460/GCN-HĐĐĐ). Online informed consent was obtained from all 1,305 participants who chose a “Yes” answer to the question, “*Do you agree to participate in this research?*”. This survey stopped when a person chose a “No” answer. All study procedures and methods were performed in accordance with the relevant regulations and guidelines.

## Results

### General characteristics of participants

Among 1,317 Vietnamese people approached, 1,305 people agreed to participate in this research (99.1%). Most participants (*n* = 1,217, 93.3%) were under 50 years old (average: 29.91 ± 11.36 years old). A third were males (*n* = 451, 34.6%). Two-fifths got married (*n* = 514, 39.4%). More than half lived in the north (*n* = 737, 56.5%). Four-fifths came from urban areas (*n* = 1,046, 80.2%). Nearly a third have an education level of university or higher (*n* = 410, 31.4%). The number of participants working in healthcare was 82 people (6.3%). The average monthly income, allowance, and/or retirement pension of about three-quarters (*n* = 977, 74.9%) were lower than twelve million Vietnam dongs (590.328US$). Almost all participants possessed at least one health insurance card (*n* = 1,275, 97.7%). About a sixth (*n* = 234, 17.9%) came down with at least one chronic disease (for example, diabetes mellitus, hypertension, and sinusitis) (Table 1).

**Table 1:** Main characteristics of participants (*n* = 1,305 people).

No	Characteristics		Number	%
1	Sex	Male	451	34.6
		Female	854	65.4
2	Age (years old)	19 to 29	819	62.8
		30 to 39	216	16.6
		40 to 49	182	13.9
		50 or above	88	6.7
3	Marital status	Married	514	39.4
		Unmarried	791	60.6
4	Region	North	737	56.5
		Central	360	27.6
		South	208	15.9
5	Area	Urban	1,046	80.2
		Rural	259	19.8
6	Highest level of education	College or lower	895	68.6
		University or higher	410	31.4
7	Working/Studying	Working in the medical field	82	6.3
		Non-healthcare jobs or students	1,223	93.7
8	Average monthly income, allowance, or retirement pension (million Vietnam dongs). Exchange rate: one million Vietnam dongs = 49.194US$.	< 6	742	56.9
		6 to < 12	235	18.0
		12 to < 18	146	11.2
		18 or higher	182	13.9
9	Having a health insurance card	Yes	1,275	97.7
		No	30	2.3
10	Contracting at least one chronic disease	Yes	234	17.9
		No	1,071	82.1
11	The frequency of Internet usage	Rarely	73	5.6
		Sometimes	287	22.0
		Usually	945	72.4
12	The frequency of online shopping (any products or services)	Never/rarely	721	55.2
		Sometimes	431	33.0
		Usually	153	11.7
13	Using the Internet to seek health information	Yes	1,273	97.5
		No	32	2.5
14	Self-diagnosis without visiting a doctor based on health information from the Internet	Yes	839	64.3
		No	466	35.7
15	Self-medication without consulting a pharmacist or doctor based on information from the Internet	Yes	953	73.0
		No	352	27.0
16	Knowledge about purchasing medicines and functional foods online (mean score ± standard deviation)		6.21 ± 2.79	
17	Attitudes towards purchasing medicines and functional foods online (mean score ± standard deviation)		87.46 ± 16.05	

Regarding Internet usage, the frequency of using the Internet among all participants was high (usually: *n* = 945, 72.4%), while that of online shopping for any products/services was relatively low (usually: *n* = 153, 11.7%). On average, a participant spent roughly 6.26 ± 3.59 h per day using the Internet. The Internet was a common source for almost all participants to seek health information (*n* = 1,273, 97.5%), including self-diagnosis (*n* = 839, 64.3%) and self-medication (*n* = 953, 73.0%). In addition, the average knowledge score of all participants regarding purchasing medicines/functional foods online was 6.21 ± 2.79 (range: 0–16). Their average attitude score was 87.46 ± 16.05 (range: 23–115) (Table 1).

### Purchasing medicines and functional foods online in the past year

**Figure 1:**
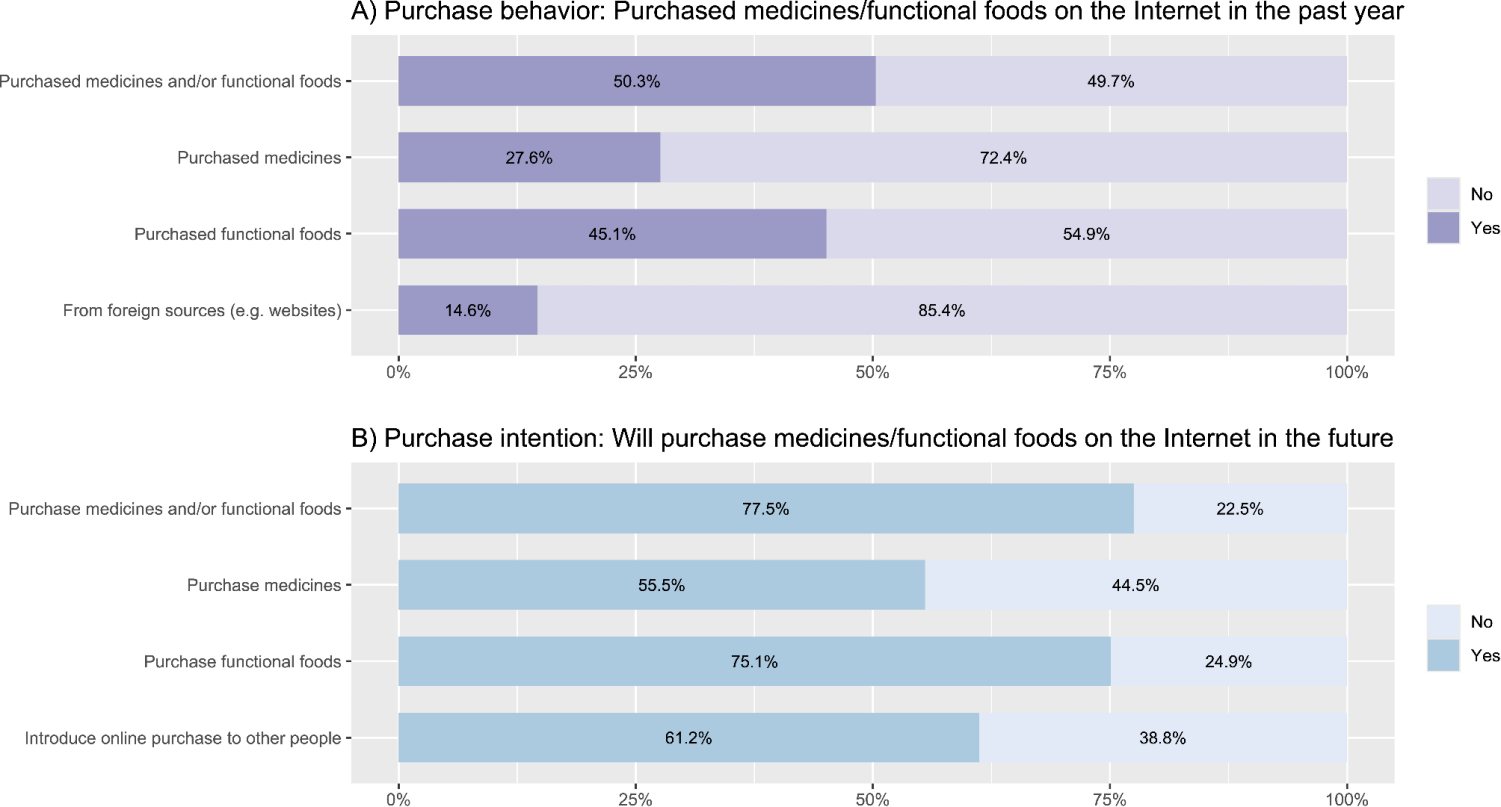
Vietnamese people’s behavior and future intention to purchase medicines and functional food online. (**A**) Purchase behavior: Purchased medicines/functional foods on the Internet in the past year, (**B**) Purchase intention: Will purchase medicines/functional foods on the Internet in the future.

In the past year, half of the participants (*n* = 656, 50.3%) purchased at least one kind of medicine and/or functional foods on the Internet (medicines: *n* = 360, 27.6%, functional foods: *n* = 588, 45.1%). One-seventh (*n* = 190, 14.6%) bought these products from foreign sources, such as international websites (Fig. 1). Among 656 buyers, nearly a third bought these products more than three times in the past year (*n* = 209, 31.9%). Participants’ points of purchase encompassed online pharmacies (*n* = 317, 48.3%), social networks (*n* = 309, 47.1%), and manufacturers’ websites (*n* = 262, 39.9%). Their sources of information included the Internet, social networks, mobile applications (*n* = 450, 68.6%), and their family/friends (*n* = 267, 40.7%). Several criteria for choosing the points to purchase medicines/functional foods online included the reviews/comments of previous buyers (*n* = 439, 66.9%), the clarity and reliability of products and manufacturers (*n* = 430, 65.5%), and the qualification and reliability of the sellers/suppliers (*n* = 426, 64.9%) In addition, 267 individuals felt satisfied with their previous experience in purchasing these products online (40.7%). The number of dissatisfied customers was 39 people (5.9%). (Table 2).

**Table 2:** Vietnamese people’s activities involving their online purchase of medicines and functional foods in the past year (*n* = 656 buyers).

No	Characteristics		Number	%
1	Purchased medicines and/or functional foods online in the past year (times)	1 to 3	447	68.1
		> 3	209	31.9
2	Point of purchase *(multiple choices)*	Online pharmacies	317	48.3
		Social networks (Facebook, Zalo…)	309	47.1
		Websites of manufacturers	262	39.9
		E-commerce websites	58	8.8
3	From where did you know medicines and functional foods can be purchased online? *(multiple choices)*	Internet, social networks, mobile applications	450	68.6
		Family, friends	267	40.7
		Healthcare providers (doctors, pharmacists…)	167	25.5
		Mass media (television, radio…)	73	11.1
4	Criteria for choosing the points to purchase medicines and functional foods online *(multiple choices)*	Reviews/comments of previous buyers	439	66.9
		The information about products and manufacturers is precise and reliable.	430	65.5
		Sellers or suppliers are reliable and have high qualifications	426	64.9
		The availability and various kinds of products	341	52.0
		Low/affordable prices	272	41.5
5	When purchasing medicines on the Internet, you did … (*n* = 360 medicine buyers) *(multiple choices)*	Make a conversation with a doctor or pharmacist before purchasing products	158	43.9
		Submit your prescription to the seller	83	23.1
		Complete a survey or answer a questionnaire about your health status	66	18.3
		Nothing of the above	118	32.8
6	Level of satisfaction for previous experiences in the online purchasing of medicines/functional foods	Completely dissatisfied	17	2.6
		Dissatisfied	22	3.4
		Neutral/Normal	350	53.4
		Satisfied	189	28.8
		Completely satisfied	78	11.9

As per the multivariate logistic regression models, factors associated with this purchase behavior of Vietnamese people included sex, marital status, education level, average time of Internet use per day, frequency of online shopping, and using the Internet to seek health information for self-diagnosis and self-medication (*p* < 0.001). The prevalence of purchasing medicines/functional foods online was lower among males (adjusted odds ratio (aOR) = 0.61, 95%CI: 0.47–0.80) and the unmarried (aOR = 0.41, 95%CI: 0.26–0.66). By contrast, this purchase behavior was more prevalent among individuals with an education level of university or higher (aOR = 2.27, 95%CI: 1.62–3.18), sometimes/usually shopping online (sometimes: aOR = 2.03, 95%CI: 1.54–2.66; usually: aOR = 3.24, 95%CI: 2.12–4.95), longer time of Internet use per day (one-hour increase: aOR = 1.08, 95%CI: 1.04–1.12), using the Internet for self-diagnosis (aOR = 1.91, 95%CI: 1.44–2.54) and self-medication (aOR = 1.79, 95%CI: 1.30–2.47) (Table 3). Univariate analyses were reported in Table S2.

**Table 3:** Factors associated with the purchase behavior of Vietnamese people regarding medicines and functional foods on the internet in the past year (multivariate analyses).

Independent variables	Purchased medicines online		Purchased functional foods online		Purchased medicines and/or functional foods online	
	aOR (95% CI)	*p*-value	aOR (95% CI)	*p*-value	aOR (95% CI)	*p*-value
1. Age (years old)			0.98 (0.96–0.99)	0.009	0.98 (0.96-1.00)	0.028
2. Sex (ref: Female)						
Male			0.56 (0.43–0.73)	< 0.001	0.61 (0.47–0.80)	< 0.001
3. Area (ref: Rural)						
Urban					1.56 (1.12–2.15)	0.008
4. Marital status (ref: Married)						
Unmarried			0.40 (0.25–0.63)	< 0.001	0.41 (0.26–0.66)	< 0.001
5. Monthly income/allowance/pension (ref: 18 or higher) (unit: million Vietnam dongs. Exchange rate: 1 million Vietnam dongs = 49.194 US$)						
< 18			0.51 (0.33–0.78)	0.002	0.59 (0.38–0.93)	0.022
6. Education level (ref: College or lower)						
University or higher	2.06 (1.57–2.70)	< 0.001	2.25 (1.62–3.13)	< 0.001	2.27 (1.62–3.18)	< 0.001
7. Average time of Internet use per day (hours)			1.06 (1.02–1.10)	0.004	1.08 (1.04–1.12)	< 0.001
8. The frequency of online shopping (ref: Rarely or never)						
Sometimes	1.72 (1.30–2.28)	< 0.001	2.00 (1.52–2.62)	< 0.001	2.03 (1.54–2.66)	< 0.001
Usually	2.99 (2.04–4.39)	< 0.001	3.24 (2.16–4.88)	< 0.001	3.24 (2.12–4.95)	< 0.001
9. Using the Internet for self-diagnosis (ref: No)	1.93 (1.40–2.66)	< 0.001	1.79 (1.35–2.37)	< 0.001	1.91 (1.44–2.54)	< 0.001
10. Using the Internet for self-medication (ref: No)	2.33 (1.61–3.39)	< 0.001	1.74 (1.26–2.41)	< 0.001	1.79 (1.30–2.47)	< 0.001
11. Attitudes towards purchasing medicines and functional foods online	0.98 (0.98–0.99)	< 0.001			0.99 (0.98-1.00)	0.022
*The Hosmer–Lemeshow test*	X-squared = 2.242, df = 8, p-value = 0.973		X-squared = 9.926, df = 8, p-value = 0.270		X-squared = 15.405, df = 8, p-value = 0.052	
*The area under the curve (AUC)*	0.700 (0.668–0.731)		0.748 (0.721–0.774)		0.754 (0.728–0.780)	

*ref* reference, *aOR* adjusted odds ratio, *95% CI* 95% confidence interval

#### Intention to purchase medicines and functional foods online in the future

More than three-quarters of the participants (*n* = 1,011, 77.5%) had the intention of purchasing medicines/functional foods online in the future (medicines: *n* = 724, 55.5%; functional foods: *n* = 980, 75.1%) (Fig. 1). Their purchase intention was associated with previous experiences in online purchases, area, contracting chronic diseases, and using the Internet for self-medication (*p* < 0.001). In particular, future purchase intention was more prevalent among people purchasing medicines/functional foods online in the past year (aOR = 13.48, 95%CI: 7.86–23.13), living in urban areas (aOR = 2.23, 95%CI: 1.56–3.17), and self-medicating via health information sought from the Internet (aOR = 1.93, 95%CI: 1.35–2.74). However, the likelihood of purchasing these products online in the future sharply diminished among those who experienced dissatisfaction with previous online purchases (aOR = 0.08, 95%CI: 0.03–0.18) and those having at least one chronic disease (aOR = 0.49, 95%CI: 0.33–0.73) (Table 4).

**Table 4:** Factors associated with future intention to purchase medicines and functional foods on the internet among Vietnamese people (multivariate analyses).

Independent variables	Purchase medicines online		Purchase functional foods online		Purchase medicines and/or functional foods online		Introduction to other people	
	aOR (95% CI)	*p*-value	aOR (95% CI)	*p*-value	aOR (95% CI)	*p*-value	aOR (95% CI)	*p*-value
1. Age (years old)	0.97 (0.95–0.99)	0.001	0.98 (0.97–0.99)	0.001				
2. Sex (ref: Female)								
Male	1.65 (1.26–2.14)	< 0.001						
3. Region (ref: Central/North)								
South			0.51 (0.34–0.75)	< 0.001	0.58 (0.38–0.88)	0.011		
4. Area (ref: Rural)								
Urban			1.83 (1.29–2.59)	< 0.001	2.23 (1.56–3.17)	< 0.001	1.56 (1.13–2.15)	0.006
5. Marital status (ref: Married)								
Unmarried	0.50 (0.31–0.80)	0.004						
6. Education level (ref: College or lower)								
University or higher	1.87 (1.35–2.60)	< 0.001	1.72 (1.18–2.52)	0.005			1.96 (1.42–2.69)	< 0.001
7. Working (ref: Healthcare)								
Non-healthcare or students	2.33 (1.39–3.90)	0.001	2.14 (1.22–3.76)	0.008	2.13 (1.19–3.83)	0.011	2.69 (1.55–4.69)	< 0.001
8. Contracting at least one chronic disease (ref: No)					0.49 (0.33–0.73)	< 0.001		
9. Average time of Internet use per day (hours)	1.05 (1.01–1.08)	0.019						
10. Times of purchasing medicines and/or functional foods online in the past year							1.32 (1.15–1.52)	< 0.001
11. Purchased medicines and/or functional foods online in the past year (ref: No)	2.53 (1.89–3.41)	< 0.001	10.91 (7.33–16.24)	< 0.001	13.48 (7.86–23.13)	< 0.001	2.89 (1.85–4.50)	< 0.001
12. Level of satisfaction								
Satisfied	1.92 (1.29–2.86)	0.001			3.68 (1.04–12.97)	0.043		
Dissatisfied	0.42 (0.21–0.85)	0.016	0.08 (0.04–0.17)	< 0.001	0.08 (0.03–0.18)	< 0.001	0.13 (0.06–0.27)	< 0.001
13. Using the Internet for self-diagnosis (ref: No)	1.61 (1.21–2.13)	< 0.001			1.53 (1.09–2.15)	0.015	1.61 (1.19–2.16)	0.002
14. Using the Internet for self-medication (ref: No)	1.66 (1.21–2.26)	0.001	2.34 (1.71–3.19)	< 0.001	1.93 (1.35–2.74)	< 0.001	1.68 (1.22–2.33)	0.002
15. Knowledge about purchasing medicines and functional foods online					1.07 (1.01–1.14)	0.022		
*The Hosmer–Lemeshow test*	X-squared = 14.726, df = 8, p-value = 0.065		X-squared = 4.128, df = 8, p-value = 0.845		X-squared = 2.629, df = 8, p-value = 0.955		X-squared = 12.547, df = 8, p-value = 0.128	
*The area under the curve (AUC)*	0.748 (0.722–0.774)		0.822 (0.797–0.847)		0.846 (0.823–0.868)		0.807 (0.784–0.830)	

*ref* reference, *aOR* adjusted odds ratio, *95% CI* 95% confidence interval

About 61.2% of participants (*n* = 799) intended to introduce the online purchase of these products to other people (Fig. 1). This future intention was significantly associated with their education level, occupation, and previous experiences in online purchases (*p* < 0.001). The intention to introduce the online purchase of medicines/functional foods to other people was more prevalent among participants having an education level of university or higher (aOR = 1.96, 95%CI: 1.42–2.69) and those not working in the medical field/being students (aOR = 2.69, 95%CI: 1.55–4.69). Furthermore, this intention was significantly associated with the online purchasing behavior in the past year (aOR = 2.89, 95%CI: 1.85–4.50) and times of purchasing medicines/functional foods online in the past year (one-purchase increase: aOR = 1.32, 95%CI: 1.15–1.52). By contrast, this intention considerably decreased among purchasers dissatisfied with previous online buying (aOR = 0.13, 95%CI: 0.06–0.27) (Table 4). Univariate analyses were reported in Table S3.

## Discussion

The development of cutting-edge technology and the omnipresence of electronic gadgets connected to the Internet have facilitated the outstanding growth of online trading in recent years. Numerous people have a predilection for online shopping because of its benefits, such as convenience and time-saving. During the COVID-19 pandemic, online trading and doorstep delivery played a crucial role in hindering the transmission of this virus and protecting people’s health. In this study, half of the participants purchased medicines and/or functional foods online in the past year. The common points of purchase included online pharmacies, social networks, and manufacturers’ websites. Factors associated with their purchase behavior included sex, marital status, education level, average time of Internet use per day, frequency of online shopping, and using the Internet to seek health information for self-diagnosis and self-medication. More than three-quarters of the participants intended to purchase these products online in the future. Factors significantly associated with this intention included their previous experiences in online purchases, area, contracting chronic diseases, and using the Internet for self-medication. Besides, three-fifths of the participants intended to introduce this buying to other people. This intention was significantly associated with their education level, occupation, and previous experiences in online purchases.

The percentage of people purchasing health products online varied among countries and years of study. A multinational web-based survey in 22 countries showed that the percentages of the participants buying medicinal products, herbal products, and supplementary/nutritional products via the Internet were 11.9%, 18.8%, and 28.8%, respectively^28^. About 14.5% of Internet users in the United States purchased a medication or vitamin online^11^. In Jordan, roughly 11.8% of people purchased medicines over the Internet in the past^32^. In this study, 50.3% of Vietnamese people purchased at least one kind of medicine and/or functional foods online (medicines: 27.6%, functional foods: 45.1%). This high percentage may spring from the time of study. People were asked about whether or not they purchased medicines/functional foods online in the past year. During this period, several outbreaks of the COVID-19 pandemic occurred in Vietnam. People were recommended not to go outside their houses. During several specific intervals, lockdowns, social distance, and self-isolation were also applied, and this pandemic became the rationale behind the online purchase of these products among many citizens. Our results were in line with the findings of several previous studies. In 2020–2023, the percentages of people in four countries (including the Czech Republic, Poland, Hungary, and Slovakia) buying medicines and other health products over the Internet were 55.5% and 63.0%, respectively^29^. In Saudi Arabia, 60.2% of people purchased products from online pharmacies in the past (within the past three months (the year 2022): 53.4%)^33^.

Sex, marital status, education level, average time of Internet use per day, frequency of online shopping, and using the Internet to seek health information were factors significantly associated with the purchase behaviors of Vietnamese people regarding medicines/functional foods on the Internet. This behavior among Vietnamese females was more popular than among males, similar to a study in Russia^34^. Another previous study even showed that the female gender was one of seven predictors of online shopping addiction^35^. A previous study showed that married people spent significantly less time on leisure activities than single individuals^36^. Online shopping can be a valuable method to save time for the former. Vietnamese people with high education levels were more likely to purchase these products online, aligning with the results of two studies in the United Arab Emirates and Romania^12,14^. Besides, the more time a person spends accessing the Internet and searching for health information, the more likely they are to reach links, websites, or online pharmacies selling medicines/functional foods. This can be a potential rationale behind the high likelihood of purchasing these products online among individuals usually shopping online, having longer time of Internet use per day, and seeking health information on the Internet for self-diagnosis and self-medication.

This study also witnessed a high proportion of Vietnamese people intending to purchase medicines/functional foods online in the future and introduce this buying to other people. Their intentions were significantly associated with previous experiences in buying these products online. Notably, the percentage of Vietnamese people dissatisfied with their previous online purchases was low, in line with a study in Saudi Arabia^33^. In addition, individuals purchasing medicines/functional foods online in the past year were 13.48 times more likely to continue buying these merchandise online in the future compared to the non-buyer group. These can facilitate the development of activities involving trading medicines and functional foods online. Several other factors also associated with Vietnamese people’s intention to purchase these products online included the frequency of Internet use/time of Internet use, the frequency of online shopping, and using the Internet to seek health information, in line with the results of a study in Hungary when individuals using the Internet more and buying goods online would be more likely to purchase medications online^24^.

There is a high potential for future uptake of the online medicines/functional foods trade. A myriad of people, not only in Vietnam but also in many other countries, intend to purchase these products online in the future^23,31,37^. Several rationales behind the great need for this online buying include convenience^38^, privacy^39^, low prices^25^, low availability of essential medicines in local pharmacies^26,40,41^, a wide variety of products, and easy online access^15,33^. Therefore, the number of people supporting the legalization of online medical product trade is on the increase^30,42,43^. The rapid proliferation of online trade, the anonymity, and the inability to enforce against overseas sellers have challenged the government and authorities to manage and control the online trading of these products^44^. Online sellers can take advantage of the lack of knowledge and perception to defraud consumers^45^. On the Internet, illegal medicine sellers usually do not require prescriptions, do not show their contact details, do not limit the number of purchasable products, do not offer pharmacists’ consultations, and even trade counterfeit and substandard medicines^9,10,46^. Up to 2023, trading medicines online is illegal in Vietnam, and no specific laws/documents relevant to the online trade of these products have been promulgated. However, the Vietnam Ministry of Health, policymakers, and relevant authorities are going to promulgate legal documents and laws related to online pharmacies and trading medicines on the Internet in the forthcoming years. Then, enhancing people’s knowledge about the online purchase of these products and how to choose legitimate points of purchase is of paramount importance as it is impossible to curb the online trading of these products.

This is the first study conducted to investigate Vietnamese people’s behavior and future intention to purchase medicines/functional foods online. Having a large sample size, employing the Bayesian Model Averaging method to select variables in the multivariate models, and the high values of AUC are other strengths. However, this study also has several following limitations. First, this is only a cross-sectional study. Therefore, the causal relationship between people’s behavior and intention to purchase medicines/functional foods online and independent factors cannot be determined. The participants cannot be representative of the Vietnamese population as they were recruited using convenience and snowballing sampling methods. In addition, using an online survey and a self-report questionnaire to collect data may lead to several biases (such as recall bias).

## Conclusions

In the past year, half of the participants purchased medicines/functional foods on the Internet. Factors associated with their purchase behavior included sex, marital status, education level, average time of Internet use per day, frequency of online shopping, and using the Internet to seek health information for self-diagnosis and self-medication. Numerous people intend to purchase these products online in the future and will introduce this online purchase to other people. The former was significantly associated with their previous experiences in online purchases, area, contracting chronic diseases, and using the Internet for self-medication, while education level, occupation, and previous experiences were associated with the latter.

## Supplementary Information

Below is the link to the electronic supplementary material.

Supplementary Material 1

## Data Availability

The datasets used and/or analyzed during the current study are available from the corresponding author upon reasonable request.

## Abbreviations

95%CI: 95% Confidence Interval
aOR: adjusted odds ratio
AUC: area under the curve
COVID-19: Coronavirus disease 2019
ICC: intraclass correlation coefficient
US: the United States
