# Supplementary material for: Vietnamese people’s behavior and future intention to purchase medicines and functional foods on the internet: a cross-sectional study
Source: Sci Rep. 2024 Oct 16;14:24267. doi: 10.1038/s41598-024-75029-5 (PMC11484985; doi:10.1038/s41598-024-75029-5)
Supplement: Supplementary file 1 — Supplementary Material 1 [file 41598_2024_75029_MOESM1_ESM.pdf]

Vietnamese people's behavior and future intention to purchase medicines and functional foods on the Internet: a cross-sectional study

Dung Anh Doan, Nhung Hong Vu, Phuong Lan Nguyen, Dai Xuan Dinh*

* Corresponding author: daidinh.hup@gmail.com. ORCID ID: 0000-0001-6387-2496.

Affiliation: Faculty of Pharmaceutical Management and Economics, Hanoi University of Pharmacy, Hanoi, Vietnam.

Table S1. Knowledge and attitude questions about purchasing medicines and functional foods on the Internet

	Statement/Item	Score				
		Totally disagree	Disagree	Normal, neutral	Agree	Totally agree
**A**	**Knowledge part**					
1	Medicines and functional foods can be purchased on the Internet (online).	0	0	0	1	2
2	There are other reputable sources to buy medicines and functional foods besides pharmacies in the hospitals and the community.	0	0	0	1	2
3	It is necessary to check the information (such as qualification and reputation) of the sellers/providers before online buying.	0	0	0	1	2
4	Nearly every medicine and functional food can be purchased on the Internet.	0	0	0	1	2
5	It is necessary to consult with healthcare professionals (such as doctors and pharmacists) before buying medicines online, especially prescription-only medicines.	0	0	0	1	2
6	Purchasing prescription-only medicines online without a prescription is legal and appropriate.	2	1	0	0	0
7	Trading medicines online can go beyond the scope of a country, causing many difficulties and challenges in management.	0	0	0	1	2
8	Selling medicines online is legal in Vietnam.	2	1	0	0	0

	Statement/Item	Score				
		Totally disagree	Disagree	Normal, neutral	Agree	Totally agree
**B**	**Attitude part**					
	Potential advantages of purchasing medicines and functional foods on the Internet					
1	Wide range of products (can buy products unavailable or not sold at nearby local hospitals or community pharmacies)	1	2	3	4	5
2	In comparison with buying products in community pharmacies, I can get more information when buying online	1	2	3	4	5
3	Affordable, lower prices, many offers, or discounts	1	2	3	4	5
4	When buying online, it is faster and easier to compare the information and prices of products than when directly buying in pharmacies	1	2	3	4	5
5	Able to order and buy products after opening hours (available 24/7)	1	2	3	4	5
6	More privacy and anonymity	1	2	3	4	5
7	Convenience (for example, doorstep delivery; in conditions of bad weather, pandemics, or epidemics; elimination of barriers for people with disabilities and senior citizens)	1	2	3	4	5
8	Easily check the availability of products (reduce the time spent traveling between pharmacies to check)	1	2	3	4	5
9	Freedom from location (can purchase products from other countries)	1	2	3	4	5
10	No waiting time or queuing at pharmacies	1	2	3	4	5
	Potential disadvantages of purchasing medicines and functional foods on the Internet					
11	Increasing the risks of polypharmacy and drug interactions	1	2	3	4	5
12	Can purchase expired, counterfeit, and/or substandard products	1	2	3	4	5
13	Information about products on the Internet may be inaccurate	1	2	3	4	5
14	Long delivery time	1	2	3	4	5

	Statement/Item	Score				
		Totally disagree	Disagree	Normal, neutral	Agree	Totally agree
15	Increasing the risks of personal information leakage and money transactions	1	2	3	4	5
16	Not getting the right products (mistakes in packing or delivery)	1	2	3	4	5
17	Lack of supervision of the authorities (such as the seller may not be licensed to sell medicines)	1	2	3	4	5
18	Difficult to choose the appropriate product due to the significant number of products on the Internet	1	2	3	4	5
19	Products can be stored/preserved under substandard conditions	1	2	3	4	5
20	The sellers may not be precisely identified (names, qualifications...)	1	2	3	4	5
21	Increasing the risks of drug abuse, self-medication, and treatment non-adherence	1	2	3	4	5
22	People under 18 years old can purchase medicines without restrictions	1	2	3	4	5
23	Difficult to distinguish between legal (registered) online pharmacies and illegal (unlicensed) commercial websites	1	2	3	4	5

Table S2. Factors associated with the purchase behavior of Vietnamese people regarding medicines and functional foods on the Internet (univariate analyses)

Independent variables	Purchased medicines online		Purchased functional foods online		Purchased medicines and/or functional foods online	
	OR (95% CI)	p-value	OR (95% CI)	p-value	OR (95% CI)	p-value
**1. Age** (years old) (continuous variable)	1.01 (1.00 - 1.02)	0.304	1.01 (1.00 - 1.02)	0.194	1.00 (0.99 - 1.01)	0.611
**2. Sex** (ref: Female)						
Male	0.85 (0.66 - 1.10)	0.220	0.54 (0.43 - 0.68)	<0.001	0.59 (0.47 - 0.74)	<0.001
**3. Region** (ref: Central)						
North	1.24 (0.92 - 1.65)	0.153	1.74 (1.34 - 2.25)	<0.001	1.73 (1.34 - 2.23)	<0.001
South	1.55 (1.06 - 2.26)	0.023	1.22 (0.86 - 1.73)	0.269	1.46 (1.04 - 2.06)	0.031
**4. Area** (ref: Rural)						
Urban	1.53 (1.10 - 2.12)	0.011	1.78 (1.34 - 2.36)	<0.001	2.08 (1.57 - 2.76)	<0.001
**5. Marital status** (ref: Married)						
Unmarried	0.74 (0.58 - 0.94)	0.015	0.51 (0.41 - 0.64)	<0.001	0.58 (0.46 - 0.72)	<0.001
**6. Education level** (ref: College or lower)						
University or higher	1.85 (1.44 - 2.38)	<0.001	3.00 (2.35 - 3.82)	<0.001	2.97 (2.32 - 3.81)	<0.001
**7. Working** (ref: Healthcare)						
Non-healthcare or being a student	1.38 (0.81 - 2.36)	0.240	1.00 (0.64 - 1.56)	0.990	1.12 (0.72 - 1.76)	0.613
**8. Income/allowance/pension** (unit: million Vietnam dongs) (ref: 12 to <18)						
<6	0.85 (0.57 - 1.27)	0.422	0.50 (0.35 - 0.72)	<0.001	0.50 (0.35 - 0.72)	<0.001
6 to <12	1.27 (0.80 - 2.00)	0.308	0.71 (0.47 - 1.07)	0.104	0.72 (0.47 - 1.09)	0.123
18 or higher	1.44 (0.89 - 2.31)	0.134	1.91 (1.21 - 3.00)	0.005	1.84 (1.16 - 2.93)	0.010
**9. Having a health insurance card** (ref: No)	1.54 (0.62 - 3.79)	0.350	1.43 (0.67 - 3.02)	0.352	1.53 (0.73 - 3.20)	0.258

Independent variables	Purchased medicines online		Purchased functional foods online		Purchased medicines and/or functional foods online	
	OR (95% CI)	p-value	OR (95% CI)	p-value	OR (95% CI)	p-value
**10. Contracting at least one chronic disease** (ref: No)	1.30 (0.96 - 1.77)	0.092	1.03 (0.78 - 1.37)	0.820	0.95 (0.71 - 1.26)	0.705
**11. Frequency of Internet use** (ref: Rarely)						
Sometimes	1.25 (0.69 - 2.28)	0.465	2.16 (1.23 - 3.80)	0.007	2.04 (1.18 - 3.52)	0.010
Usually	1.28 (0.73 - 2.24)	0.393	2.30 (1.35 - 3.91)	0.002	2.38 (1.43 - 3.97)	<0.001
**12. Average time of Internet use per day** (hours) (continuous variable)	1.06 (1.03 - 1.10)	<0.001	1.08 (1.04 - 1.11)	<0.001	1.10 (1.07 - 1.14)	<0.001
**13. Frequency of online shopping** (ref: Rarely or never)						
Sometimes	1.88 (1.44 - 2.47)	<0.001	2.27 (1.78 - 2.90)	<0.001	2.38 (1.86 - 3.03)	<0.001
Usually	3.35 (2.33 - 4.83)	<0.001	4.02 (2.77 - 5.84)	<0.001	4.23 (2.87 - 6.23)	<0.001
**14. Using the Internet to seek health information** (ref: No)	3.77 (1.14 - 12.44)	0.030	8.22 (2.49 - 27.12)	<0.001	5.65 (2.16 - 14.77)	<0.001
**15. Using the Internet for self-diagnosis** (ref: No)	2.57 (1.94 - 3.41)	<0.001	2.20 (1.74 - 2.79)	<0.001	2.30 (1.82 - 2.90)	<0.001
**16. Using the Internet for self-medication** (ref: No)	2.83 (2.04 - 3.93)	<0.001	1.90 (1.47 - 2.45)	<0.001	1.97 (1.53 - 2.53)	<0.001
**17. Knowledge about purchasing medicines and functional foods online** (continuous variable)	0.98 (0.93 - 1.02)	0.266	1.00 (0.96 - 1.04)	0.958	0.99 (0.96 - 1.03)	0.725
**18. Attitudes towards purchasing medicines and functional foods online** (continuous variable)	0.99 (0.98 - 1.00)	0.015	1.00 (0.99 - 1.01)	0.882	1.00 (0.99 - 1.00)	0.347
ref: reference, OR: odds ratio, 95%CI: 95% confidence interval.						

Table S3. Factors associated with the future intention to purchase medicines and functional foods on the Internet among Vietnamese people (univariate analyses)

Independent variables	Purchase medicines		Purchase functional foods		Purchase medicines and/or functional foods		Introduction to other people	
	OR (95% CI)	p-value	OR (95% CI)	p-value	OR (95% CI)	p-value	OR (95% CI)	p-value
**1. Age** (years old) (continuous variable)	1.00 (0.99 - 1.01)	0.491	0.98 (0.97 - 0.99)	<0.001	0.98 (0.97 - 0.99)	<0.001	1.00 (0.99 - 1.01)	0.901
**2. Sex** (ref: Female)								
Male	1.24 (0.99 - 1.57)	0.065	0.72 (0.55 - 0.93)	0.012	0.72 (0.55 - 0.94)	0.016	0.97 (0.77 - 1.23)	0.799
**3. Region** (ref: Central)								
North	1.81 (1.40 - 2.34)	<0.001	1.14 (0.85 - 1.52)	0.391	1.19 (0.88 - 1.60)	0.260	1.60 (1.24 - 2.07)	<0.001
South	1.86 (1.32 - 2.63)	<0.001	0.74 (0.51 - 1.09)	0.125	0.90 (0.60 - 1.33)	0.581	1.04 (0.74 - 1.47)	0.809
**4. Area** (ref: Rural)								
Urban	1.81 (1.38 - 2.39)	<0.001	2.56 (1.92 - 3.42)	<0.001	2.76 (2.06 - 3.71)	<0.001	2.27 (1.73 - 3.00)	<0.001
**5. Marital status** (ref: Married)								
Unmarried	0.69 (0.55 - 0.87)	0.002	1.03 (0.80 - 1.34)	0.794	1.18 (0.91 - 1.54)	0.212	0.71 (0.56 - 0.89)	0.003
**6. Education level** (ref: College or lower)								
University or higher	2.22 (1.74 - 2.84)	<0.001	2.07 (1.54 - 2.80)	<0.001	1.92 (1.41 - 2.61)	<0.001	2.53 (1.95 - 3.28)	<0.001
**7. Working** (ref: Healthcare)								
Non-healthcare or being a student	1.73 (1.10 - 2.72)	0.017	1.81 (1.14 - 2.90)	0.013	1.98 (1.23 - 3.17)	0.005	2.12 (1.35 - 3.33)	0.001
**8. Income/allowance/pension** (unit: million Vietnam dongs) (ref: 12 to <18)								
<6	0.51 (0.35 - 0.74)	<0.001	0.45 (0.28 - 0.73)	0.001	0.50 (0.30 - 0.83)	0.007	0.37 (0.25 - 0.56)	<0.001
6 to <12	0.57 (0.37 - 0.88)	0.011	0.36 (0.21 - 0.61)	<0.001	0.37 (0.21 - 0.63)	<0.001	0.38 (0.24 - 0.61)	<0.001
18 or higher	1.26 (0.79 - 2.02)	0.331	0.96 (0.52 - 1.79)	0.908	1.00 (0.53 - 1.88)	0.992	1.28 (0.75 - 2.17)	0.371
**9. Having a health insurance card** (ref: No)	2.19 (1.03 - 4.64)	0.041	2.05 (0.98 - 4.30)	0.058	2.03 (0.95 - 4.31)	0.066	2.10 (1.01 - 4.37)	0.046

Independent variables	Purchase medicines		Purchase functional foods		Purchase medicines and/or functional foods		Introduction to other people	
	OR (95% CI)	p-value	OR (95% CI)	p-value	OR (95% CI)	p-value	OR (95% CI)	p-value
**10. Contracting at least one chronic disease** (ref: No)	0.94 (0.71 - 1.25)	0.682	0.68 (0.50 - 0.93)	0.014	0.65 (0.48 - 0.90)	0.009	0.91 (0.68 - 1.22)	0.527
**11. Frequency of Internet use** (ref: Rarely)								
Sometimes	1.69 (1.00 - 2.87)	0.051	2.14 (1.27 - 3.61)	0.004	2.08 (1.23 - 3.51)	0.006	2.27 (1.34 - 3.84)	0.002
Usually	2.41 (1.48 - 3.95)	<0.001	3.95 (2.43 - 6.42)	<0.001	4.17 (2.56 - 6.79)	<0.001	2.83 (1.74 - 4.63)	<0.001
**12. Average time of Internet use per day** (hours) (continuous variable)	1.08 (1.04 - 1.11)	<0.001	1.07 (1.03 - 1.11)	<0.001	1.10 (1.05 - 1.14)	<0.001	1.06 (1.02 - 1.09)	0.001
**13. Frequency of online shopping** (ref: Rarely or never)								
Sometimes	1.67 (1.31 - 2.13)	<0.001	2.29 (1.70 - 3.07)	<0.001	2.27 (1.67 - 3.09)	<0.001	1.83 (1.42 - 2.35)	<0.001
Usually	2.44 (1.68 - 3.55)	<0.001	3.35 (2.02 - 5.55)	<0.001	3.08 (1.84 - 5.17)	<0.001	2.14 (1.46 - 3.13)	<0.001
**14. Purchased medicines and/or functional foods on the Internet in the past year** (ref: No)	3.93 (3.12 - 4.95)	<0.001	9.17 (6.59 - 12.75)	<0.001	12.70 (8.65 - 18.64)	<0.001	6.89 (5.35 - 8.89)	<0.001
**15. Times of purchasing medicines and/or functional foods online in the past year** (continuous variable)	1.29 (1.21 - 1.37)	<0.001	1.46 (1.33 - 1.60)	<0.001	1.53 (1.38 - 1.70)	<0.001	1.85 (1.68 - 2.04)	<0.001
**16. Level of satisfaction** (ref: Neutral/Normal)								
Never purchased	0.32 (0.24 - 0.42)	<0.001	0.10 (0.06 - 0.15)	<0.001	0.07 (0.04 - 0.12)	<0.001	0.16 (0.11 - 0.21)	<0.001
Satisfied	2.21 (1.51 - 3.23)	<0.001	2.11 (0.96 - 4.62)	0.062	4.22 (1.22 - 14.62)	0.023	1.91 (1.19 - 3.05)	0.007
Dissatisfied	0.42 (0.22 - 0.83)	0.012	0.11 (0.05 - 0.23)	<0.001	0.09 (0.04 - 0.20)	<0.001	0.18 (0.09 - 0.36)	<0.001
**17. Using the Internet to seek health information** (ref: No)	4.61 (1.98 - 10.73)	<0.001	8.22 (3.76 - 17.95)	<0.001	9.45 (4.32 - 20.66)	<0.001	4.18 (1.92 - 9.11)	<0.001

Independent variables	Purchase medicines		Purchase functional foods		Purchase medicines and/or functional foods		Introduction to other people	
	OR (95% CI)	p-value	OR (95% CI)	p-value	OR (95% CI)	p-value	OR (95% CI)	p-value
**18. Using the Internet for self-diagnosis** (ref: No)	2.40 (1.91 - 3.03)	<0.001	2.53 (1.96 - 3.28)	<0.001	2.75 (2.11 - 3.59)	<0.001	2.46 (1.94 - 3.10)	<0.001
**19. Using the Internet for self-medication** (ref: No)	2.17 (1.70 - 2.79)	<0.001	2.74 (2.10 - 3.58)	<0.001	2.87 (2.18 - 3.78)	<0.001	2.25 (1.76 - 2.89)	<0.001
**20. Knowledge about purchasing medicines and functional foods online** (continuous variable)	1.05 (1.01 - 1.09)	0.027	1.05 (1.00 - 1.10)	0.042	1.06 (1.01 - 1.11)	0.018	1.04 (0.99 - 1.08)	0.088
**21. Attitudes towards purchasing medicines and functional foods online** (continuous variable)	1.00 (1.00 - 1.01)	0.361	1.01 (1.00 - 1.02)	0.016	1.01 (1.00 - 1.02)	0.027	1.01 (1.00 - 1.01)	0.078

ref: reference, OR: odds ratio, 95%CI: 95% confidence interval.
